# Cord Entanglement in Monochorionic Diamniotic Twin Pregnancy: A Rare Diagnostic and Clinical Challenge

**DOI:** 10.7759/cureus.82412

**Published:** 2025-04-17

**Authors:** Snigdha Kumari, Abhay Kumar, Vishali Thapa

**Affiliations:** 1 Obstetrics and Gynecology, Postgraduate Institute of Medical Education and Research, Chandigarh, IND

**Keywords:** cord entanglement, intrauterine fetal demise, monochorionic diamniotic twins, prenatal ultrasound, true knots

## Abstract

Umbilical cord entanglement is a well-known complication in monochorionic monoamniotic twin pregnancies but is extremely rare in monochorionic diamniotic (MCDA) pregnancies due to the presence of an intertwin membrane. We report a case of a 24-year-old G4P1-0-2-1 at 33 weeks and two days of gestation presenting with intrauterine fetal demise of both twins in an MCDA pregnancy. Clinical evaluation and ultrasonography confirmed the demise, and postpartum examination revealed cord entanglement with true knots despite an intact intertwin membrane. This case highlights the importance of vigilant sonographic surveillance of the intertwin membrane and umbilical cords in high-risk MCDA pregnancies to prevent adverse outcomes.

## Introduction

Monochorionic diamniotic (MCDA) twin pregnancies are characterized by two fetuses sharing a single placenta but being separated by an intertwin membrane. Cord entanglement is a well-recognized risk in monochorionic monoamniotic (MCMA) twins but is considered rare in MCDA twins due to the protective membrane barrier. However, reports suggest that spontaneous septostomy or preterm premature rupture of membranes (PPROM) can lead to functional monoamnionicity, increasing the risk of cord entanglement [1,2]. This case report details an unusual instance of cord entanglement in an MCDA pregnancy with an intact intertwin membrane, emphasizing the need for careful prenatal monitoring.

## Case presentation

**Figure 1:**
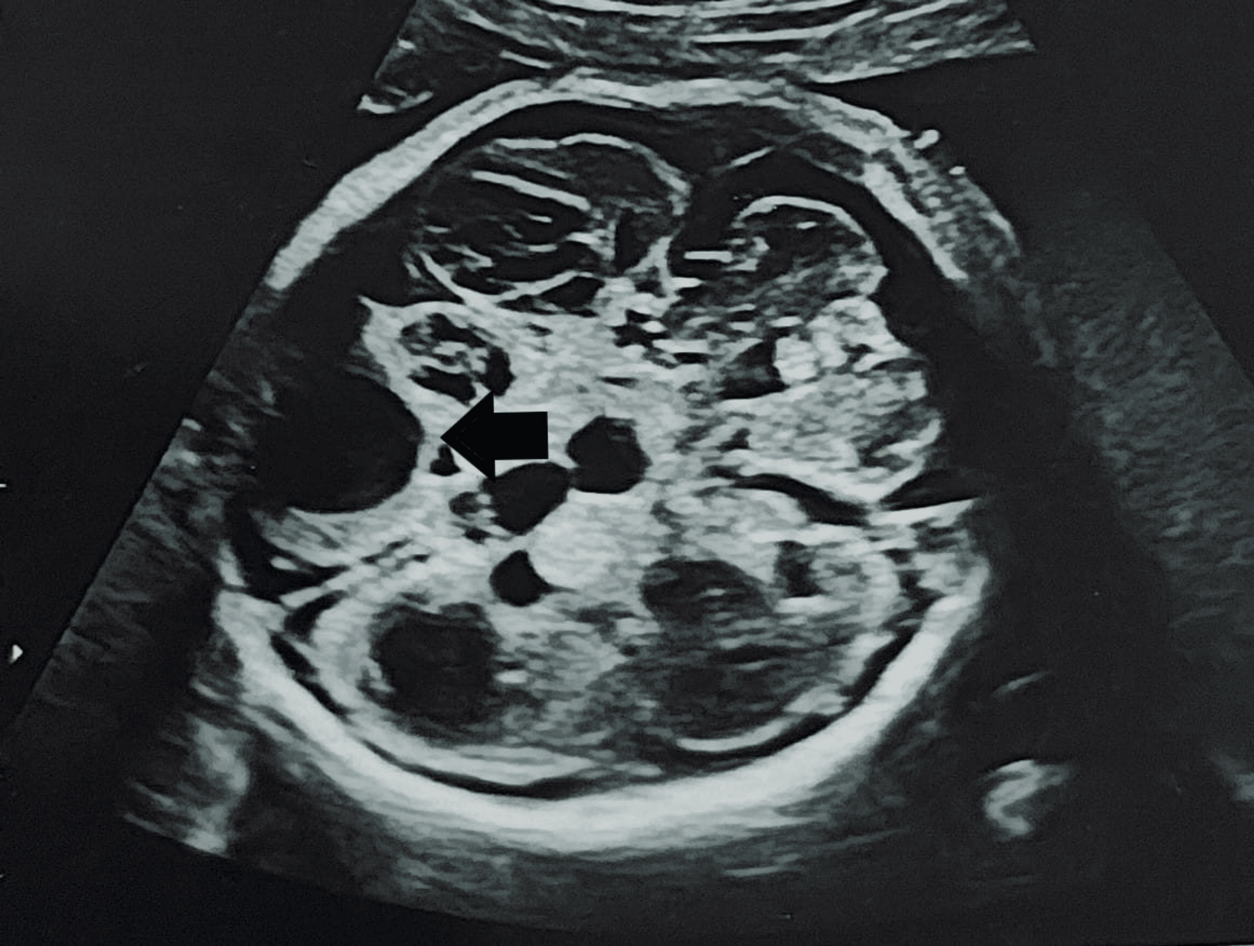
Bilateral dilated ventricles (arrow)

**Figure 2:**
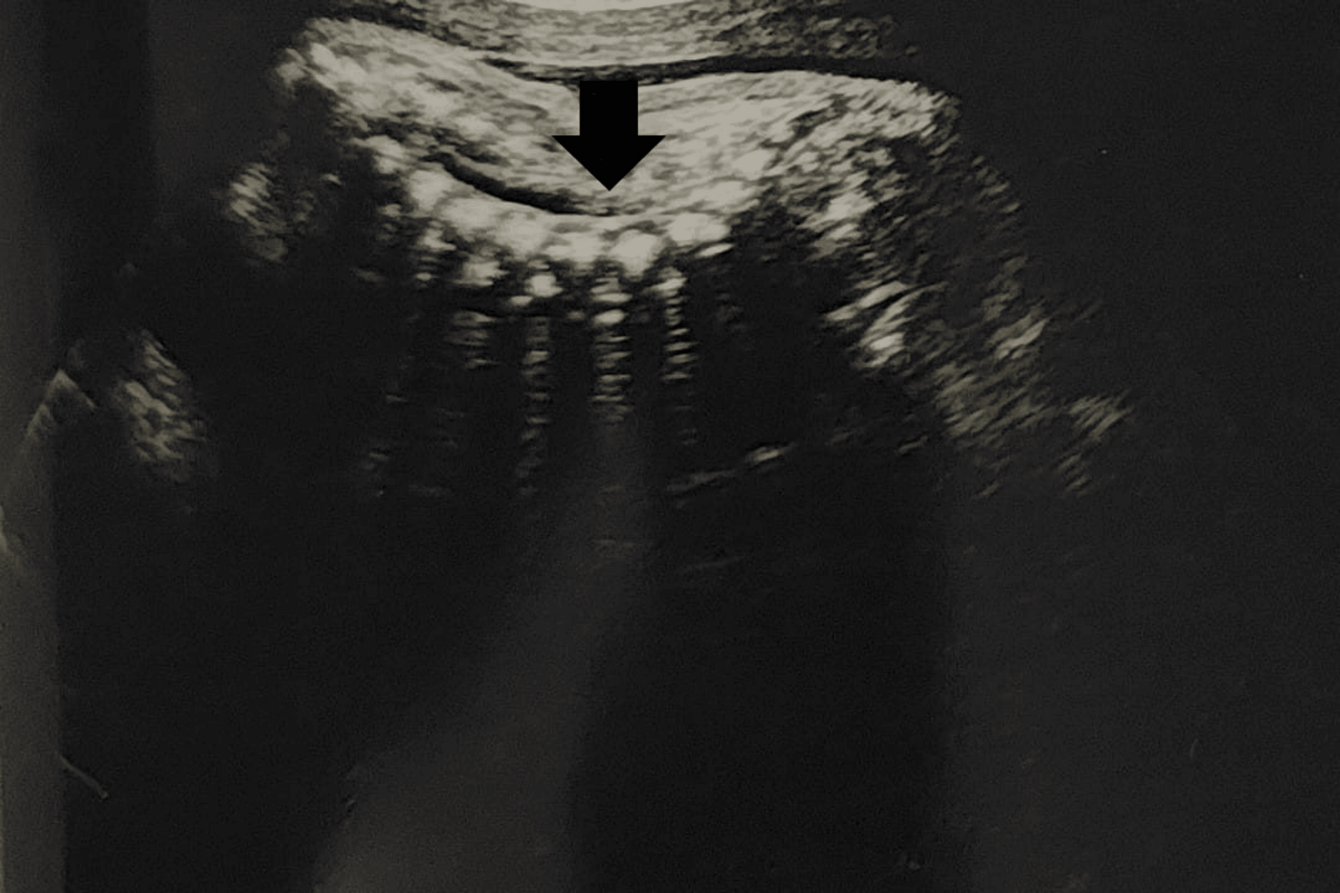
Lordosis of the dorsolumbar spine (arrow)

We present the case of a 24-year-old G4P1-0-2-1 at 33 weeks and two days of gestation with an MCDA twin pregnancy, presenting in latent labor with intrauterine fetal demise of both twins. She was nondiabetic and nonhypertensive, with no history of vaginal bleeding. Clinical examination revealed stable vital signs and an overdistended and irritable uterus, with multiple fetal parts palpable. Per vaginal examination revealed cervical dilation of 2 cm. Fetal heart sounds could not be auscultated with a stethoscope. Emergency ultrasound confirmed the demise of both twins (Spalding sign positive), with a single posterior placenta and no retroplacental clot. Multiple antenatal ultrasounds had been performed (Table 1), and at the 31 weeks and six days scan, Twin B showed cystic encephalomalacia, bilateral dilated ventricles (Figure 1), lordosis of the dorsolumbar spine (Figure 2), and arthrogryposis of the upper and lower limbs.

**Table 1:** Ultrasound findings for MCDA twin pregnancy AFI, amniotic fluid index; CMF, congenital malformations; EFW, estimated fetal weight; MCDA, monochorionic diamniotic; MSK, musculoskeletal; NB, nasal bone; NT, nuchal translucency

Period of gestation	Twin A findings	Twin B findings	Additional notes
14 weeks (MCDA)	NT: 1.2 mm; NB: present	NT: 1.5 mm; NB: present	NT/NB screening performed
19+1 weeks (MCDA)	Prominent renal pelvis: right 4.4 mm, left 2.2 mm; AFI: 25 cm; EFW: 219 g	Urinary bladder collapsed; liquor volume adequate; EFW: 161 g; no gross CMF	Onset of slight discordance
27+4 weeks	EFW: 962 g; AFI: 23 cm	EFW: 612 g; AFI: 15 cm; 29% growth discrepancy	Significant growth discordance noted
31+6 weeks	EFW: 1,902 g	EFW: 874 g; cystic encephalomalacia; bilateral ventriculomegaly; dorsolumbar spine lordosis; limb arthrogryposis	Severe CNS and MSK anomalies

**Figure 3:**
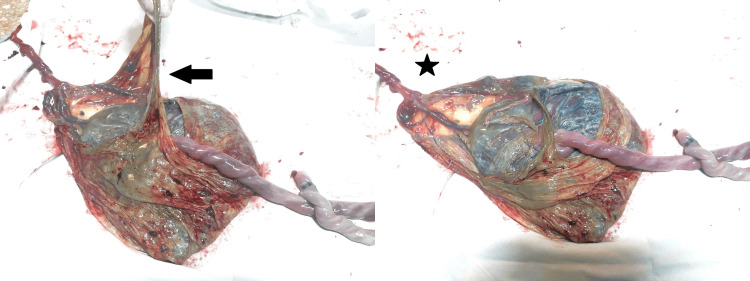
Examination of the placenta revealed an intact intertwin membrane (arrow) with Twin A cord attachment adjacent to the intertwin membrane and a thinner Twin B cord having velamentous attachment (star)

**Figure 4:**
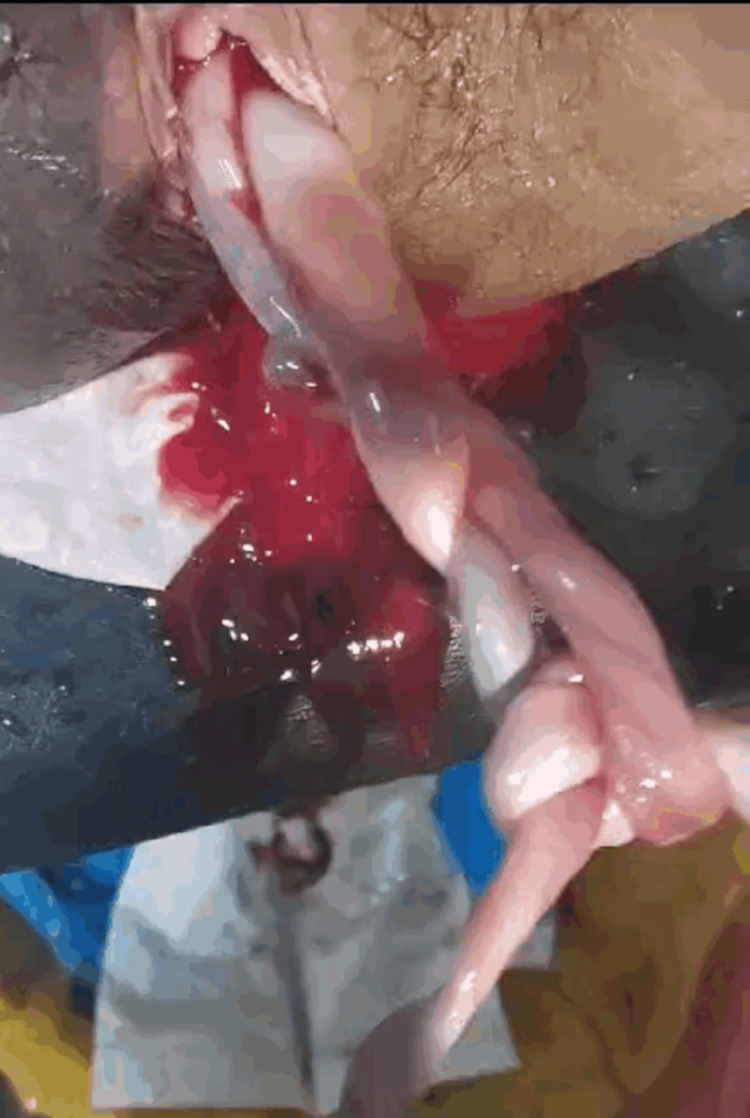
Cord entanglement with two loose true knots in the umbilical cord

Her quadruple screen and fetal echocardiogram were normal. The patient went into spontaneous labor, and during delivery, amniotomy of both twins was done separately. She delivered macerated twin male fetuses: Twin A weighed 1,542 g and Twin B 1,072 g, with no externally visible malformations due to autolysis. Post-delivery examination of the placenta revealed an intact intertwin membrane and velamentous cord insertion for Twin B (Figure 3). Cord entanglement with two loose true knots was observed (Figure 4).

## Discussion

Monochorionic twin gestations are at a higher risk for perinatal complications such as preterm delivery, twin-to-twin transfusion syndrome, intrauterine growth restriction, and umbilical cord knots and entanglement [3]. True knots of the umbilical cord occur in approximately 1% of deliveries and may lead to asphyxia and fetal demise; however, antepartum management remains undetermined [4]. The most significant predisposing factor is MCMA twin gestation. Although rare in MCDA twins, literature reports its occurrence following spontaneous septostomy, PPROM, or invasive intrauterine procedures [5,6]. Once the intertwin membrane ruptures, an MCDA gestation becomes functionally monoamniotic, with perinatal mortality rates of up to 70% and a greater than 50% chance of cord entanglement [7].

In our case, no evidence of septostomy or PPROM was identified, making this an unusual presentation of cord entanglement despite an intact membrane. The pathophysiology remains unclear; a likely explanation for the cord entanglement in this case is the presence of a velamentous cord insertion in the smaller twin. In such cases, the umbilical vessels run unprotected through the amniotic membrane for a considerable distance before forming the actual cord. It is possible that these exposed vessels extended into the amniotic sac of the larger twin, leading to entanglement between the cords. Cord entanglement may initially be loose; however, it has the potential to tighten and compromise fetal circulation later in pregnancy [8].

Due to these associations, a thorough evaluation of the intertwin membrane and umbilical cords at each sonographic assessment is advised. Umbilical cord insertion anomalies are more common in twins compared to singletons. Velamentous cord insertion occurs in 2% of singletons, 7% of dichorionic, and 12% of monochorionic twin pregnancies [9] and is associated with unequal placental territory sharing and discordant growth.

True knots can have variable presentations, ranging from asymptomatic to poor fetal outcomes. Pregnancies with true knots have a more than fourfold increased risk of stillbirth [10]. True knots are uncommon and difficult to diagnose during pregnancy; they resemble a four-leaf clover on ultrasonography, but this pattern is nonspecific and can also be seen with false knots or closely spaced loops of the umbilical cord [11].

Despite advancements in prenatal imaging, visualizing cord entanglement remains a challenge in MCDA twins. Doppler ultrasound may aid in identifying abnormal cord positioning and compromised blood flow, offering potential early indicators of fetal distress.

Given the high perinatal mortality associated with cord entanglement, routine monitoring of membrane integrity and umbilical cord configuration is crucial, especially in high-risk MCDA pregnancies. Early detection and timely intervention - including hospitalization and close fetal surveillance - may improve outcomes.

## Conclusions

This case highlights a rare event of cord entanglement in an MCDA twin pregnancy despite an intact intertwin membrane, resulting in intrauterine fetal demise. It underscores the need for meticulous ultrasound evaluation of the intertwin membrane and umbilical cords in high-risk MCDA pregnancies. Further research is warranted to determine the incidence and contributing risk factors for cord entanglement and to develop preventive antenatal management strategies. Improved imaging may help enhance early detection and intervention for such cord-related complications.
